# Supplementary material for: Comparison of Phenolic and Flavonoid Compound Profiles and Antioxidant and α-Glucosidase Inhibition Properties of Cultivated Soybean (Glycine max) and Wild Soybean (Glycine soja)
Source: Plants (Basel). 2021 Apr 20;10(4):813. doi: 10.3390/plants10040813 (PMC8074397; doi:10.3390/plants10040813)
Supplement: Supplementary file 1 [file plants-10-00813-s001.zip › plants-1172512-supplementary.pdf]

plants

MDPI

Supplementary Figure

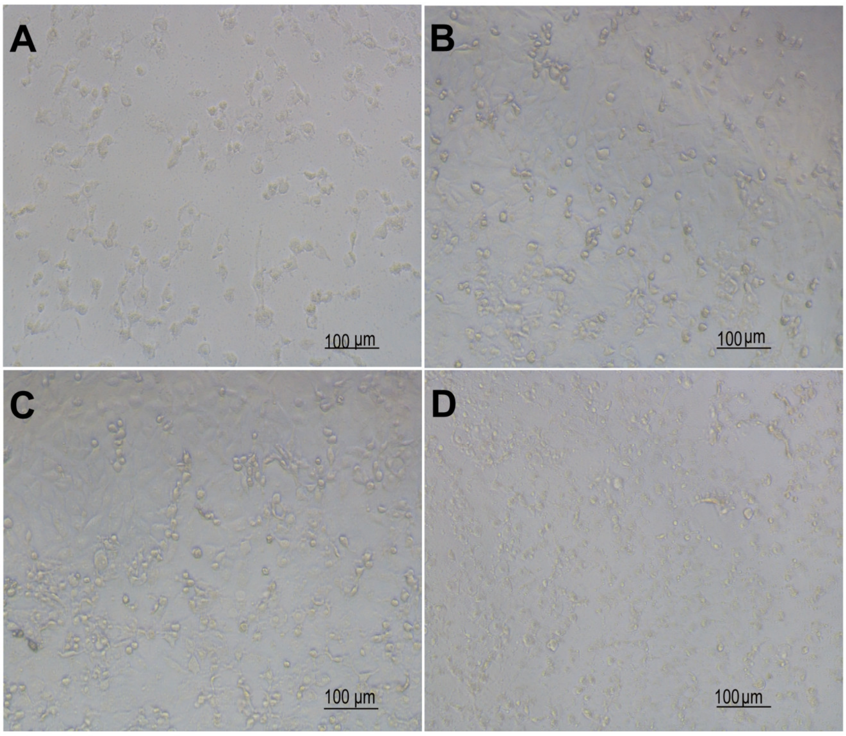

Fig. S1. Cell morphology of human endothelial cell following H_2O_2 stimulation (A), acetylcysteine (B), inhibition rate after pre-incubation with WS (C) and CS (D).
